# Decoding Prosodic Information from Motion Capture Data: The Gravity of Co-Speech Gestures

**DOI:** 10.1162/opmi_a_00196

**Published:** 2025-04-29

**Authors:** Jacob P. Momsen, Seana Coulson

**Affiliations:** Joint Doctoral Program in Language and Communication Disorders, San Diego State University and UC San Diego; Yale Child Study Center, Yale University, New Haven, Connecticut, USA; Cognitive Science Department, UC San Diego

**Keywords:** speech, gesture, kinetics, machine learning, prosody

## Abstract

In part due to correspondence in time, seeing how a speaking body moves can impact how speech is apprehended. Despite this, little is known about whether and which specific kinematic features of co-speech movements are relevant for their integration with speech. The current study uses machine learning techniques to investigate how co-speech gestures can be quantified to model vocal acoustics within an individual speaker. Specifically, we address whether kinetic descriptions of human movement are relevant for modeling their relationship with speech in time. To test this, we apply experimental manipulations that either highlight or obscure the relationship between co-speech movement kinematics and downward gravitational acceleration. Across two experiments, we provide evidence that quantifying co-speech movement as a function of its anisotropic relation to downward gravitational forces improves how well those co-speech movements can be used to predict prosodic dimensions of speech, as represented by the low-pass envelope. This study supports theoretical perspectives that invoke biomechanics to help explain speech-gesture synchrony and offers motivation for further behavioral or neuroimaging work investigating audiovisual integration and/or biological motion perception in the context of multimodal discourse.

## INTRODUCTION

Speech and co-speech gestures are typically considered the unified output of a single communicative system due to their close temporal coordination (e.g., McNeill, 1995). Moreover, prosodic aspects of speech, e.g., amplitude and pitch measures, have been shown to serve as anchor points for how co-speech gestures are designed in time (Leonard & Cummins, 2011; Wagner et al., 2014). Here we examine evidence for the gesture-speech physics hypothesis that gesture-speech synchrony results in part from biomechanical constraints on speech production (Pouw, Harrison, et al., 2020b) and explore its potential implications for the comprehension of multimodal discourse. Moreover, we test the plausibility of the sensorimotor account of multimodal prosody (SAMP) that suggests the vestibular system plays an important role in the timing of the body movements accompanying speech (Momsen & Coulson, in press).

Work exploring the timing relations between speech and co-speech gestures has primarily focused on movements made with the upper limbs—a canonical example being “beat” gestures, often identified as downward flicks of the hand or arm, such that the moment of maximum energy expenditure near the end of the stroke tends to coincide with peaks in spoken intonation or stress patterns (McNeill, 1995; Tuite, 1993). A pulse-like quality is a hallmark feature of these simple, emphatic movements, and it is also observable in gestures that serve other communicative functions, such as deictic or iconic gestures (e.g., Fung & Mok, 2018; Rochet-Capellan et al., 2008). Investigating the phenomena of multimodal prosody, most researchers have assumed the synchrony between speech and gestures is explained exclusively by neurocognitive factors (e.g., de Ruiter, 2000).

By contrast, Pouw and colleagues have suggested that speech-gesture synchrony derives in no small part from the kinetic features of gesture, i.e., the underlying physical causes of movement, as well as their collateral influence across distal body parts (Pouw & Fuchs, 2022). Acoustic delivery during phonation is susceptible to self- or externally- generated changes in full body motion, which often require anticipatory muscle activations in the trunk and legs to ensure postural stability. Co-speech gestures with varying velocity signatures can transfer kinetic energy to the respiratory system, resulting in the modulation of the pitch and amplitude of the speech signal (Pouw, Harrison, et al., 2020b). In this way, momentum generated by co-speech gestures can impose direct constraints on acoustic parameters of the speech signal.

Beyond its implications for production, the biophysical relationship between speech and gestures suggests novel hypotheses about how movements of the speaking body influence the comprehension of speech in multimodal discourse. In addition to gestures’ documented enhancements to semantic processing (see, e.g., Wu & Coulson, 2011), the causal influence of body movement on the acoustics of speech suggests co-speech gestures provide listeners with visual cues for prosodic processing. On this account, a beat gesture is not simply a communicatively relevant movement of the hand or arm, but an implicit cue for impending movement of the torso, neck, and head due to momentum transfer.

Our sensorimotor account of multimodal prosody (SAMP) suggests that audiences implicitly contextualize a speaking body in terms of force-related variables to capture these more global, biomechanical interactions. The present study aims to test some basic premises of this account. First, SAMP suggests that gestures in multimodal discourse are informative for the amplitude of upcoming speech. Moreover, it suggests gestures might be encoded in terms of their kinetic properties above and beyond their kinematic ones. Although kinetic and kinematic descriptions of movement are complementary, they are nevertheless distinct conceptually and numerically. Kinematics describes the motion of objects, whereas kinetics describes the forces and torques that cause motion. One primary distinction between the two classes of descriptions is the treatment of physical mass. Though irrelevant for the calculation of kinematic variables such as velocity and acceleration, mass is a key component of kinetic variables such as kinetic energy and momentum. Further, any kind of inference about the kinetic properties of movement during the apprehension of multimodal speech would require incorporating information about the environmental force fields in which those movements occur, i.e., gravity.

### The Current Study

The current study was designed to test the relevance of kinetic variables for modeling associations between speech and gesture by exploiting the fact that gravity dictates the way movement-related forces operate along the earth-vertical plane.

By quantifying movement data, machine learning algorithms can be trained to fit a function that transforms information about those movements into numerical predictions of other systematically related time-varying signals, such as vocal acoustics as represented by a low-pass speech envelope. The current study takes this approach to identify the most relevant properties of co-speech movements for predicting prosodic elements of continuous speech. We test two hypotheses that are both motivated by the question of whether representing human movement in terms of kinetics impacts how easy it is to use those movements to predict associated vocal output. One way to approach this is by attempting to quantify the influence of gravitational constraints on co-speech movements, and test whether this affects the ability to decode the prosodic properties of the accompanying speech.

To assess this, we generated linear filters between speech envelope information and quantified movement data contained in a speech-gesture database containing over 3 hours of naturalistic discourse (Ferstl & McDonnell, 2018). This database uses a 53-marker motion capture setup, which provides position data within a 3D coordinate plane at each frame (59.94 fps). Using ridge regression to learn a Temporal Response Function (Crosse et al., 2016), we constructed models that relate various movement parameters to fluctuations in the amplitude of the speech envelope. By comparing the ability of these models to decode prosodic features of the speech, we identify features of bodily movements that are most relevant for predicting the amplitude of a speaker’s upcoming vocal output. Because the models incorporate motion capture data from several body segments, this approach also allows us to assess how informative each body part is for inferring prosodic features of the speech.

## EXPERIMENT 1

Our first experiment investigates whether the directional axis of co-speech movements impacts their relevance for predicting the speaker’s vocal output. Several descriptive analyses of co-speech gestures suggest that variations in spoken prosody tend to co-occur with the “downbeat” or extension phase of co-speech gestures (e.g., Kendon, 1980). This suggests contingencies between movement data and speech might be more evident when co-speech movements are represented in terms of their displacements along the vertical axis than displacements across the horizontal axis. Moreover, if kinetic features of body movements are systematically related to speech prosody, we might expect our estimation of prosodic features to benefit from an encoding that incorporates the influence of gravity on body movement.

To test these hypotheses, we used ridge regression to decode the amplitude of the speech envelope from separate components of the motion capture data, all using the first derivative of position information (velocity). We trained three separate models such that each model was only given information about movements along a single axis: the x-axis (horizontal), the z-axis (depth), or the y-axis (vertical). The influence of gravity on vocal acoustics suggests the decoder based on vertical movements should out-perform the decoder based on horizontal movements.

Further, the SAMP suggests that learning contingencies between movement data and speech should be more evident when movement data includes kinetic information regarding the influence of gravity. To test this, we introduced a second condition that was crossed with the 3-level Axis factor described above. Described as movement Encoding, this factor varied whether the first derivative position data was quantified using either a scalar (Speed), or a vector (Velocity). These two encodings are numerically equivalent in terms of the magnitude of the movement. Only the vector representation, however, incorporates the direction of the movement. That is, in the Speed model, movements were characterized in terms of how fast the sensors were moving, whereas the Velocity model also described their direction (left versus right in x-axis and, critically, up versus down in the y-axis).

While we do not explicitly quantify movement in terms of kinetic properties such as changes in kinetic and potential energy, gravity’s operation along the earth-vertical axis means that force dynamics for movements that occur along the y-axis will vary as a function of their direction, i.e., greater forces are required to move the arms from the waist to the head than from the head to the waist. By contrast, motion orthogonal to the gravitational plane (i.e., motion in the x-axis), does not vary in terms of the forces required to generate them. By masking or unmasking the directional information of co-speech movement kinematics, it is possible to assess whether kinetic information implicitly reflected in directional anisotropies across the vertical plane impacts how well speech can be decoded from full body motion. We thus expect a greater impact of the Encoding factor on the y-axis than the x-axis.

### Methods

#### Motion Capture.

23 BioVision Motion Capture (BVH) taken from Database I of the Trinity Speech Gesture repository were integrated with the Mocap Toolbox for MATLAB to perform all preprocessing steps of the movement data prior to the TRF analysis. Each BVH file contained, on average, 9.6 minutes of natural, unscripted discourse content from a male native English speaker speaking freely in response to several conversational prompts. The discourse content ranges from humorous storytelling to descriptions about pop-culture references, e.g., the rules and history of the speaker’s favorite sport. The speaker exhibits a relatively animated speaking style, frequently engaging in expressive co-speech gestures as well as movements of the head and trunk.

**Figure 1.**
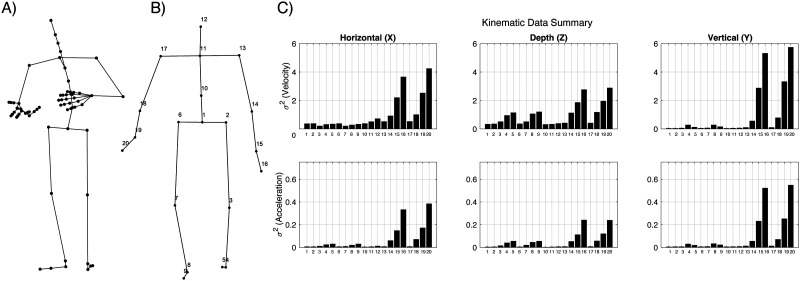
A) Motion capture model used in the original Trinity Speech-Gesture database. B) The transformed Dempster body segment model used in the Temporal Response Function analysis. C) Graphical depictions of the variance in the speaker’s movements estimated from the signal at each of the segments in the Dempster body depicted in B. These estimates utilized all database files used in the current study.

To reduce the size of the motion capture data, the original marker system was transformed into a sparser body-segment model composed of 20 markers by averaging position data across associated markers over time (Dempster’s model; see Figure 1). Kinematic variables were computed from the mean position data separately across 3 directional axes and then down sampled to 30 Hz, resulting in 20 kinematic vectors for each type of movement Encoding (Velocity and Speed).

#### Speech Processing.

To assess the prosodic fluctuations in the speech stream, we filtered the speech envelope signal from each discourse clip, as the amplitude envelope is thought to more directly capture suprasegmental features of speech that describe its prosodic structure (Myers et al., 2019). To prepare the speech data for the decoder analysis, audio files from each discourse clip were low-pass filtered at 10 Hz using the Speech Envelope and Acoustic Landmarks toolbox for MATLAB (Alexis, 2023; MacIntyre et al., 2022). The speech envelope was then resampled to 30 Hz before the decoder analysis.

#### Decoding Analysis.

Regularized linear regression was used to relate the motion capture data to the speech amplitude envelope by fitting a backward decoder model—a form of temporal response function (see Crosse et al., 2016 for computational details). The decoder model describes a multivariate linear transformation of the movement data associated with each of the 20 markers in the Dempster transformed body model to the univariate speech envelope representation. In addition to regression coefficients for each mocap marker, the decoder is also defined by a preselected time window associated with the motion capture data corresponding to the speech envelope amplitude at any individual time sample. A lagged time series was defined by a window of –1000 ms to 200 ms relative to the speech envelope, such that the decoder transformation at each time sample was described by the weighted motion signal across all body parts from 1 second prior to and 200 ms after the speech envelope. This technique automatically distributes regression weights across time and channels (mocap markers) according to their relevance for modeling variance in the speech signal. This allows us to quantify the importance of specific body segments for decoding the speaker’s vocal output.

To perform the decoder analysis, motion capture and speech data from each individual database file was partitioned into folds containing approximately 15 s of discourse information. Data from a single fold in each database file was set aside as a “test set,” while the remaining folds were used as a “training set” in a regularization procedure to optimize model generalizability. The winning decoder model determined from the optimization and training steps was then applied to the left out “test set” to render a predicted speech envelope by using the motion capture information input as the set of independent variables to the decoder function. To assess decoder performance, a Pearson correlation was computed between the predicted speech envelope and the actual speech envelope corresponding to the “test set” fold.

Correlation coefficients for each (15-s) fold across the 23 database files provided a metric for decoding performance. These coefficients were used as the dependent variable in a linear mixed effects regression analysis (LMER) to investigate the effects of direction Axis, movement Encoding, and their interaction on model performance. A stepwise model fitting procedure began with a minimal intercept-only model including a random intercept for the database file that the discourse was taken from. Likelihood ratio tests were used to identify the best-fit model. All reported p-values have been adjusted for multiple comparisons using a Bonferroni correction.

In sum, in Experiment 1 we trained 6 models on the first derivative of body position to predict speech the envelope, and assessed how the directional Axis (X, Y, Z) as well as movement Encoding (Velocity, Speed) influences the precision with which speech envelope information can be decoded from those movements of the body. Our main empirical predictions include superior decoding performance for models trained on y-axis (vertical) data relative to those trained on x- or z-axis data, as well as superior performance for models trained on movement Velocity (viz., a vector representation), compared to Speed (viz., a scalar representation). Critically, we predict that the effect of movement Encoding will be modulated by the directional axis of movement, such that the performance benefit associated with Velocity will be more robust for models trained on y-axis motion relative to models trained on x-axis data.

### Results

**Figure 2.**
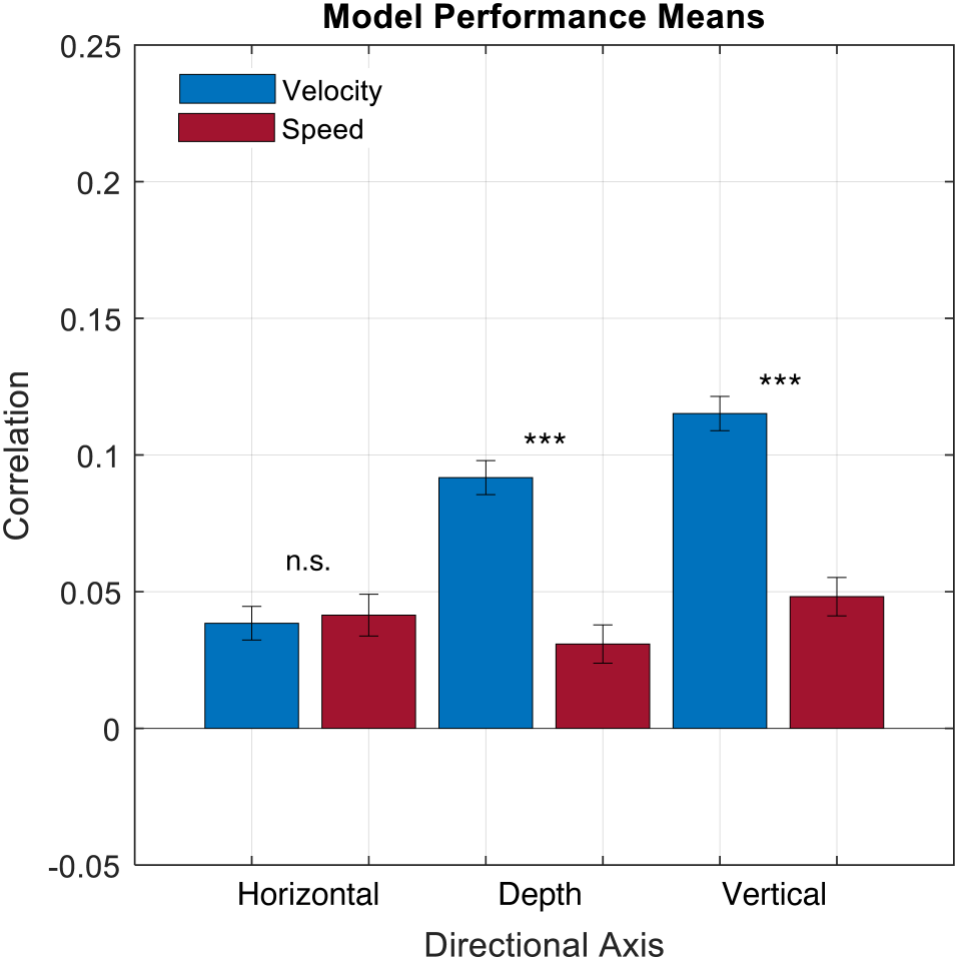
Decoder performance by Axis and movement Encoding. Model performance averaged across all database files as a function of direction Axis and movement Encoding.

The comparison of decoding performance across models trained on biological motion represented as a vector (Velocity) or a scalar (Speed) indicated a best-fit model including an interaction between direction Axis and movement Encoding (Table 1). This model revealed a significant main effect of direction Axis, such that models trained on vertical kinematic information outperformed models trained purely on either horizontal or depth information (Depth: *β* = 0.05; t = 11.1; *p* < 0.001; Vertical: *β* = 0.08; t = 16.0; *p* < 0.001; Horizontal (Intercept): *β* = 0.04; t = 7.88; *p* < 0.001). Furthermore, the interaction between Axis and movement Encoding indicated a significant reduction in performance for Speed models amongst those trained on Depth or Vertical movement (Depth: *β* = −0.06; t = −9.4; *p* < 0.001; Vertical: *β* = −0.07; t = −10.3; *p* < 0.001), but not across models trained on Horizontal movement data (*β* = 0.003; t = 0.62; p = 0.5) (Figure 2).

**Table 1.** Experiment 1 results. The model with an interaction between direction Axis and movement Encoding provides the best fit.

Model	Δ AIC	Δ Log Likelihood	*p*-value
*r* ~ 1 + (1|*File*)	–	–	
*r* ~ 1 + *Axis* + *Encoding* + (1|*File*)	−301.5	163.7	<0.001
*rr* ~ 1 + *Axis* ∗ *Encoding* + (1|*File*)	−429.6	219.8	<0.001

## EXPERIMENT 2

Experiment 1 provided initial evidence in support of the claim that when the quantification of co-speech movements bears more resemblance to their kinetic properties, they provide more useful information about the prosodic properties of associated speech compared to other salient kinematic information such as the velocity of horizontal movement in the gestures. Experiment 2 offers an alternative test of the hypothesis by exploiting a common manipulation in the biological motion literature. This involves inverting the kinematic display of a moving body across its vertical axis, which is commonly associated with impaired perception and qualitatively different neural responses relative to upright stimuli (see Troje & Westhoff, 2006 for discussion). The inversion effect has been hypothesized to reflect the way gravity-compatible spatiotemporal trajectories are critical for accurate biological motion processing. Notably, there is considerable evidence showing that learned experience with the effects of gravity influences perception and action by refining expectations about how objects naturally behave in 3-D space (Indovina et al., 2005; Jörges & López-Moliner, 2017; Lacquaniti et al., 2015). How expectations associated with gravitational forces interact with biological motion perception is less well understood, but there is indeed causal evidence suggesting that gravity plays a role in regulating the analysis of biological motion kinematics (Wang et al., 2022). We hypothesize this kinetic information may play a role in the apprehension of co-speech gestures.

Research suggests that compatibility with downward gravitational acceleration is relevant for the way auditory stimuli are integrated with biological motion signals (e.g., Brooks et al., 2007; Effenberg, 2005; Saygin et al., 2008; Shen et al., 2023), however whether this is also relevant for speech-gesture integration is currently unknown. In Experiment 2, we train speech decoders on acceleration information from the motion capture data and then test their ability to generalize to three different conditions: the standard “test set” from the left-out input, a horizontal inversion of the test set, and a vertical inversion of the test set. If gravitational information is relevant for decoding speech amplitude, performance should suffer more from vertical than horizontal inversion.

### Methods

Pre-processing steps related to the speech and motion capture data for Experiment 2 were identical to those described in Experiment 1, except for that the kinematic variables associated with each body marker were calculated as the 1st derivative of their velocity.

Rather than using unique decoder models trained on different axes, as in Experiment 1, the decoder models used in Experiment 2 were trained on acceleration information from all 20 marker locations and all 3 directional axes. Thus 60 input vectors were used to decode speech envelope information within each database file and data fold.

After model training, regression weights from the decoder were given 3 forms of “left out” kinematic information that comprised the factor body Orientation: preserved, horizontal inversion, and vertical inversion. Predicted speech envelopes rendered from the preserved Orientation condition were generated from the same speaker kinematics that the models were originally trained on. Test data associated with the horizontal inversion condition was created by inverting the directional information reflected in the acceleration vectors along the horizontal axis, viz., by multiplying them by −1. Test sets from the vertical inversion condition were similarly created by inverting the directional information associated with acceleration trajectories in the vertical plane.

This resulted in 3 sets of correlation coefficients from the same set of models, differing only with respect to the Orientation of the kinematics that were used to generate predicted envelope information. We expected superior performance for the test set using preserved kinematic signatures compared to both types of inverted test sets. In addition, we predicted a significant reduction in model performance when tested on vertically inverted kinematics, relative to horizontally inverted kinematics.

### Results

Prediction performance was influenced by kinematic differences due to the body Orientation of the test set, as indicated by a significant improvement in model fit after the addition of the Orientation factor relative to an intercept-only model (see Table 2). The effect of body Orientation reflected reduced prediction performance for horizontally and vertically inverted kinematics relative to the preserved body orientation the models were originally trained on (Horizontal Inversion: *β* = −0.06; t = −15.1; *p* < 0.001; Vertical inversion: *β* = −0.11; t = −26.7; *p* < 0.001). A follow-up comparison between the two inverted body orientations revealed significantly reduced performance associated with the vertical, compared to the horizontally inverted body (*β* = −0.05; t = −11.3; *p* < 0.001) (Figure 4).

**Table 2.** Experiment 2 results. The mixed effects model including body Orientation provides the best fit.

Model	Δ AIC	Δ Log Likelihood	*p*-value
*rr* ~ 1 + (1|*File*)	–	–	
*rr* ~ 1 + *Orientation* + (1|*File*)	−617.4	310.7	<0.001

## GENERAL DISCUSSION

Here we test the hypothesis that learning the relationship between prosodic features of speech and co-speech gestures is improved by estimating kinetic properties of the speaker’s movements. Experiments 1 and 2 used conditional manipulations that modified how speaker motion was quantified, with the aim of varying the degree to which kinetic information was implicitly captured by them. We found that models improved when they were trained on data that more closely approximated kinetic features of movement. The analyses in Experiment 1 indicate that a vector representation, i.e., one that retains directional information, is a superior way to encode motion along the vertical, but not horizontal, axis. Moreover, in Experiment 2, we provide evidence that learned relationships between co-speech movements and aspects of spoken prosody generalize worse to kinematics associated with vertically inverted bodies than to horizontally inverted ones. In keeping with the importance of kinetic descriptions of co-speech gestures – i.e., those that incorporate gravitational force constraints – we found support for our hypothesis that co-speech movement along the vertical axis was more informative for predicting a speaker’s vocal output than movement along the horizontal axis.

Our ability to decode changes in the intensity of the speech signal from movement parameters of the accompanying gestures indicates that those movement parameters provide insight into the prosodic features of discourse. The above-chance performance of these models is consistent with the gesture-speech physics hypothesis that the synchrony between gestures and speech acoustics arises due to biomechanical forces (Pouw & Fuchs, 2022). Noting that the production of speech engages muscles important for other aspects of movement and postural control, Pouw and colleagues have shown that movements of the arms produce a transfer of forces throughout the body that affect the respiratory system and, consequently, the acoustic properties of speech (e.g., Pouw et al., 2020a). Since there is a greater force in downwards than upwards gestures, downwards movements will have a greater impact on the respiratory system. The present study, in finding that directional encodings in the vertical plane are more relevant for speech intensity than those in the horizontal plane is thus consistent with the gesture-speech physics hypothesis. Likewise, we find the mapping between kinematic information about gestures and the amplitude of the speech envelope is more robust to left-right reversals than to vertical inversion, again reinforcing the relevance of biophysical causes for the link between gestural and vocal dimensions of prosody.

Much of the work that supports the gesture-speech physics hypothesis has utilized somewhat artificial tasks such as continuous vocalization and the rhythmic production of CV syllables (Pouw, Harrison, et al., 2020b; Pouw et al., 2020a, 2023, 2024). However, these laboratory tasks allow for careful measurement of participants’ movements and vocal acoustics that support inferences regarding the causal links between gestures and speech. For example, by asking participants to flex and extend their arms and wrists during a continuous vocalization task, Pouw et al. (2020a) showed that peaks in the amplitude and fundamental frequency of the speech signal were associated with peaks in the physical impetus of the movements. Similarly, rhythmically produced CV syllables timed to coincide with the extension of the arm have been shown to be slightly louder than those timed to coincide with the flexion of the arm, which in turn were slightly louder than those produced with no movement at all; moreover, the greater impulse arm extensions also led to more respiration-related activity, suggesting the relationship between movement and the acoustics of speech is mediated by the respiratory system (Pouw, Harrison, et al., 2020b). Finally, experimental manipulation of the mass of the arms via a one-kilogram weight leads to greater muscle activation as measured in the electromyogram (EMG) and louder peaks in the intensity of speech envelope (Pouw et al., 2023, 2024).

In showing that whole body kinetics are aligned with prosodic targets, our findings here reinforce the causal link between them and suggest it extends to more naturalistic speech production tasks. As such, results of the present study are in keeping with a growing body of research that has employed ecologically valid tasks (Pearson & Pouw, 2022; Pouw, Paxton, et al., 2020). Moreover, the continuous nature of the mapping goes beyond previous work that has noted discrete points of correlation at peaks in the envelope. Of course, this conclusion is qualified by the fact that observed effects were quite small, suggesting that movement parameters provide only partial information regarding speech amplitude. Note that while the Pearson correlation coefficients in Experiments 1 and 2 were relatively low, their magnitude compares favorably to those reported in other studies that have used the temporal response function (TRF) to model the amplitude of the speech envelope (Crosse et al., 2015; Drennan & Lalor, 2019). To our knowledge, no other study has used the TRF to examine the relationship between co-speech gesture and the speech envelope, so it is difficult to assess the overall success of these decoders.

**Figure 3.**
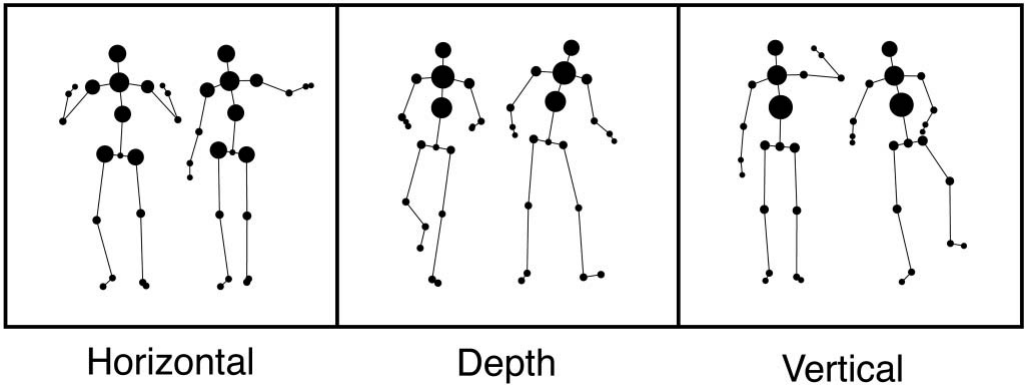
Decoder weights by body part. Mean decoder weights from the 3 Velocity models corresponding to each directional Axis are represented by the scaled size of individual markers across the depicted bodies. Absolute values of the regression coefficients corresponding to each body part in the decoding analysis were averaged across time lags to identify their relative contribution to predictions associated with the speech envelope. Across all Axes, changes in head, neck, and torso velocity held a stronger relationship with the speaker’s vocal output than movement of the upper and lower limbs.

Considering that expressive manual gestures (i.e., beats) primarily involve downward thrusts of the hand or arm, one possible objection to the current findings is that the amount of variance in the motion capture data is too sparse in some of the planes (i.e., horizontal or depth) for the regression-based decoder to identify speech-gesture correspondences. Although the summary statistics depicted in Figure 1 suggest that movement of the upper limbs (segments 13–20 in Figure 1B and 1C) was slightly more prominent within the vertical than the horizontal plane, variance associated with other body segments was comparable across the axes, with some, such as the head (segment 12) and trunk (segments 10 and 11) displaying less variance in the vertical plane than in the other two axes. As Figure 3 suggests that the amplitude of the speech envelope was more tightly yoked to movements of the head, neck, and trunk than those of the hands and arms, data sparsity does not seem to prevent the identification of mappings between movements and speech acoustics.

**Figure 4.**
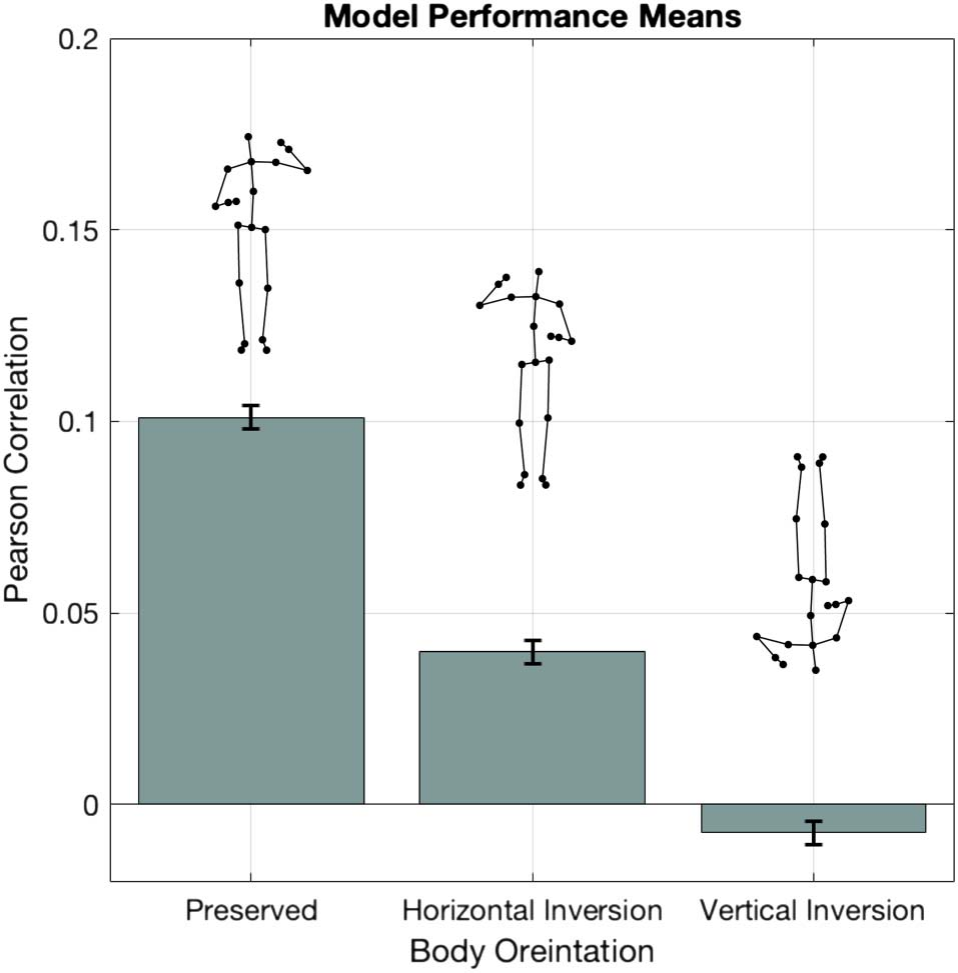
Decoder performance by body Orientation. Model performance averaged across all database files as a function of the test set used to predict speech envelope information. While both body Orientation manipulations resulted in poorer performance than the preserved body test set, kinematic signatures from a vertically inverted body were more detrimental to prediction performance than a horizontally inverted body.

In fact, although the regression-based approach used here is not as powerful as some non-linear system identification techniques (e.g., neural networks), its strength is that it yields readily interpretable weights. Figure 3 thus indicates the relative importance of movements of the head, neck, and trunk for the intensity of the speech envelope, in keeping with observational reports that highlight the coincidence of non-limb movements with prosodic events (e.g., Hadar et al., 1983). Indeed, movements across the entire body, including the head, torso, and face, are entangled with prosodic speech qualities (see Wagner et al., 2014 for a review). For example, the apex of head motion is closely correlated with peaks in the amplitude and/or pitch, particularly during phrasal boundaries (Krahmer & Swerts, 2007; Yehia et al., 2002). Research has suggested that biomechanical linkages between upper limb movement and the respiratory-vocal system can explain semiotic phenomena such as multimodal negation, in which negation words such as “not” or “never” are expressed by coordinated prosodic stress, lengthening of the initial syllable, and lateral sweep gestures (Harrison, 2024). By revealing a systematic relationship between the kinematics of co-speech movements and prosodic fluctuations in the continuous speech, results of the present study underline the validity of “visual prosody” (Biau et al., 2016; Graf et al., 2002). However, as noted elsewhere, rather than considering prosody to be an acoustic feature of speech that is augmented with gestures, we consider prosody to be inherently multimodal and encoded at least in part by the self-motion encodings in the vestibular system (Momsen & Coulson, in press).

More generally, our results are consistent with predictions from the SAMP that suggests prosodic rhythms evident in the acoustics of speech and the accompanying movements of the speaker’s body are grounded in a multimodal signal encoded in part by the vestibular system – the neural system responsible for sensing body movement and balance. Engaging in face-to-face conversation is a bodily act that involves movements of the articulators (e.g., for spoken languages, body parts like the vocal cords, tongue, lips, and teeth) along with concurrent movements of the hands, arms, trunk, and head. The SAMP suggests that the vestibular system participates in speech production due to the impact of these movements on balance. Moreover, the language comprehension system takes advantage of multimodal vestibular codes for registering the movements of the speaker’s body and coordinating dynamic attention to their speech. Findings of the current study support non-cognitive explanations for how and why expressive movements of the limbs impact speech perception (Bosker & Peeters, 2021; Pouw & Dixon, 2022).

While our data aligns with both the SAMP and the gesture-speech physics hypothesis, the SAMP makes distinct predictions that could be tested in future work. From the angle of speech production, the SAMP posits that movements of the head and body are designed in time to correct for afferent predictions made by the vestibular system (Momsen & Coulson, in press). Head movement directly stimulates the vestibular organs in the ear and thus could be used to minimize prediction error that results from the vibrotactile impact that the speaker’s speech has on their own vestibular organs. Unlike the biophysical effects that have been demonstrated for movements of the upper and lower limbs (e.g., Serré et al., 2022), head movements have less impact on the respiratory system and are thought to have relatively minor consequences for vocal acoustics (see Tiede et al., 2019 for discussion). Thus, in addition to manual co-speech gestures, the SAMP may explain the spatiotemporal properties of the head movements that often coincide with vocal behavior (Pouw et al., 2022; Tiede et al., 2019). Overall, though, gesture-speech physics and SAMP are broadly compatible as both acknowledge the important role of biomechanical factors in synchrony between gesture and speech.

Cognitive psychologists have typically assumed that synchrony between speech and gestures arises from a coordinated planning process (Wagner et al., 2014), perhaps due a specialized module for multimodal temporal processing (de Ruiter, 2000). As outlined above, both gesture-speech physics and SAMP contrast with more cognitive accounts that locate multimodal prosody in speakers’ intentions to emphasize or punctuate their speech at discrete moments – with kinetic properties of gesture being relatively inconsequential for their impact on speech processing. By contrast, the current findings locate systematic correspondences between a speaker’s movements and their vocal behavior in continuous discourse, supporting the likelihood that audiences may utilize information in biological motion dynamics that provide predictive affordances for speech perception. Once established such vocal entangled gestures can become semiotic resources that ground their meaning in the conceptual system and support pragmatic inference. As the SAMP posits that perceptual sensitivity to kinetic information critically supports multisensory integration during discourse processing, it implies that sensitivity to the environmental forces that influence movement plays a critical role in this process (Indovina et al., 2005). While sometimes overlooked, gravity acts as a ubiquitous and pervasive constraint on human movement dynamics and consequently one whose effects on vocal acoustics are registered by participants in face-to-face conversation. In conclusion, the current study suggests it may be fruitful to pursue behavioral or neuroimaging work to investigate whether and how people utilize the information that kinetic descriptions of gesture convey regarding the intensity of the speech signal in the comprehension of multimodal discourse.

## Data Availability Statement 

Code associated with this manuscript can be accessed at https://github.com/j-pohaku/TheGravityofCo-SpeechGestures. The associated data can be accessed with permission from The Trinity Speech-Gesture Dataset (https://trinityspeechgesture.scss.tcd.ie/).
